# Mechanisms of *Porphyromonas gingivalis* to translocate over the oral mucosa and other tissue barriers

**DOI:** 10.1080/20002297.2023.2205291

**Published:** 2023-04-26

**Authors:** Caroline A. de Jongh, Teun J. de Vries, Floris J. Bikker, Susan Gibbs, Bastiaan P. Krom

**Affiliations:** a Department of Preventive Dentistry, Academic Centre for Dentistry Amsterdam (ACTA), University of Amsterdam and Vrije Universiteit Amsterdam, Amsterdam, the Netherlands; b Department of Periodontology, Academic Centre for Dentistry Amsterdam (ACTA), University of Amsterdam and Vrije Universiteit Amsterdam, Amsterdam, the Netherlands; c Department of Oral Biochemistry, Academic Centre for Dentistry Amsterdam (ACTA), University of Amsterdam and Vrije Universiteit Amsterdam, Amsterdam, the Netherlands; d Department of Molecular Cell Biology and Immunology, Amsterdam UMC, Vrije Universiteit Amsterdam, Amsterdam, the Netherlands; e Department of Oral Cell Biology, Academic Centre for Dentistry Amsterdam (ACTA), Vrije Universiteit Amsterdam, Amsterdam, The Netherlands

**Keywords:** *Porphyromonas gingivalis*, oral mucosa, translocation, blood dissemination, periodontitis

## Abstract

**Introduction:**

The oral pathogen *Porphyromonas gingivalis* is not only associated with periodontitis but also with systemic diseases elsewhere in the body. The mechanisms by which *P. gingivalis* travels from the oral cavity to other organs in the body are largely unknown. This review describes the four putative mechanisms supported by experimental evidence, which enable translocation of *P. gingivalis* over the oral mucosa, endothelial barriers and subsequent dissemination into the bloodstream.

**Mechanisms:**

The first mechanism: proteolytic enzymes secreted by *P. gingivalis* degrade adhesion molecules between tissue cells, and the extracellular matrix. This weakens the structural integrity of the mucosa and allows *P*. *gingivalis* to penetrate the tissue. The second is transcytosis: bacteria actively enter tissue cells and transfer to the next layer or the extracellular space. By travelling from cell to cell, *P. gingivalis* reaches deeper structures. Thirdly, professional phagocytes take up *P. gingivalis* and travel to the bloodstream where *P. gingivalis* is released. Lastly, *P. gingivalis* can adhere to the hyphae forming Candida albicans. These hyphae can penetrate the mucosal tissue, which may allow *P. gingivalis* to reach deeper structures.

**Conclusion:**

More research could elucidate targets to inhibit *P. gingivalis* dissemination and prevent the onset of various systemic diseases.

## Introduction

The oral cavity can be colonized by more than 700 different species of microbes, including bacteria, fungi, viruses and protozoa. Together they form a complex biological system also known as the oral microbiome. This oral microbiome is the second most diverse microbiome in the human body, after the gut [1]. Several different niches can be distinguished in the oral cavity, each having their own unique microbial composition. The oral microbiome is dynamic and can be influenced by behavioral factors such as diet, smoking and stress; and by physiological changes of the host such as hormonal fluctuations and age [2,3]. When these environmental conditions change the composition and phenotypes of the microbiome also changes. This can lead to disruption of the microbial balance, or dysbiosis of the oral microbiome, characterized by the emergence of pathogenic bacteria. Dysbiosis can result in oral diseases such as caries and gingivitis. Gingivitis, a reversible inflammation of the gingiva, may progress to the more severe disease periodontitis, which is defined by alveolar bone loss and loss of clinical attachment of the periodontal ligament [4].

The major pathogens that are associated with the development of periodontitis are known as the red complex in the pyramid of Socransky. This red complex consists of three bacterial species: *Porphyromonas gingivalis*, *Treponema denticola* and *Tannerella forsythia* [5,6]. The one that is most broadly studied in relation to periodontitis is *P. gingivalis*, a Gram-negative strictly anaerobic bacterium [7]. This bacterium colonizes the gingival sulcus, predominantly found in patients suffering from periodontitis. *P. gingivalis* interacts with other oral microorganisms that colonize the gingiva in earlier stages of periodontal disease, such as *Streptococcus spp*. and *Fusobacterium nucleatum*. Interactions with these species are important for the colonization of *P. gingivalis* in the oral cavity [5]. This review is focused on *P. gingivalis*, which has a variety of virulence factors which aid in invasion or destruction of host tissue and evasion of the host immune response. Major virulence factors of *P. gingivalis* include its fimbriae and cysteine proteases known as gingipains [8]. Gingipains are essential for the survival of this proteolytic bacterium and play an important role in its pathogenicity. There are two types of gingipains: Arginine-specific gingipains (Rgp) and lysine-specific gingipains (Kgp) can cleave peptide bonds at the C-terminal after arginine and lysine amino acids, respectively. Gingipains can be secreted, expressed on the bacterial surface or released in outer membrane vesicles [9]. They are responsible for various pathogenic activities such as: evasion of host defense systems, activation of kallikrein/kinin system, lysis of fibrin and fibrinogen, and activation of the blood clotting system [10].

In addition to oral disease, periodontal pathogens such as *P. gingivalis* have been associated with various systemic diseases, including atherosclerosis, diabetes, pneumonia, rheumatoid arthritis, chronic kidney disease and Alzheimer’s disease (AD) [11–14]. *P. gingivalis* has been found in artery tissues of patients suffering from atherosclerosis and in the brains of patients with Alzheimer’s disease [15,16]. This suggests that the bacterium is able to disseminate into the bloodstream of patients and travel to various parts of the human body. In order to do so, it has to pass the oral mucosa (epithelium) and endothelium of the blood vessel. It is very likely for *P. gingivalis* to enter the bloodstream easily via bleeding gingiva, a common sign of periodontitis [17], or after tissue trauma due to dental procedures [17,18]. These mechanisms are not specific for *P. gingivalis* and would result in many different microorganisms from the oral cavity to disseminate into the blood. It was postulated that fewer different bacterial species can be found in the blood than the oral cavity [18]. For example, DNA of oral pathogens was detected in synovial fluid of patients suffering from rheumatoid arthritis which did not correspond to the amount of DNA found in dental plaque. The frequency of *Prevotella intermedia* and *P. gingivalis* DNA was almost as high in synovial fluid as in dental plaque, whereas the DNA of other species were not as frequently found in synovial fluid as in dental plaque [19]. This suggests that there is a specific mechanism for these pathogens to enter the blood and/or that these bacteria survive longer in circulation, by evading host defenses [18]. It should be emphasized that a possible passive mechanism such as bleeding should be followed up by an active translocation such as described above, crossing tissue barriers away from the periodontium, such as the brain, where *P. gingivalis* has been detected [16]. In addition, *P. gingivalis* can be found in 25% of healthy individuals not suffering from periodontitis [20]. Such a relatively high prevalence in healthy individuals should not be overlooked when considering highly prevalent diseases such as atherosclerosis and AD. Whether these healthy individuals with *P. gingivalis* also develop these diseases, has not been studied.

The exact mechanism by which *P. gingivalis* actively crosses tissue barriers is currently unknown. When considering an active role of *P. gingivalis* in invading the host tissue, it has been reported that gingipains play a crucial role in the breakdown of tissue barriers [21].

*P. gingivalis*, as well as its secreted gingipains, have been found in the brains of AD patients [16]. In the brain, the gingipains stimulate factors that are associated with AD, such as neuroinflammation, accumulation of amyloid β (Aβ) and phosphorylation of Tau protein [16,22,23]. In one of the more recent studies, mice were orally infected with *P. gingivalis* for 6 weeks and an increased amount of Aβ plaques was found in their brains. On top of this, gingipain inhibitors had a neuroprotective effect by lowering Aβ accumulation and lowering immune responses, suggesting that gingipains are involved in these processes [16]. For all these effects to occur, *P. gingivalis* would have to enter the brain and so there should be a mechanism to pass the blood–brain barrier as well as the oral mucosa.

**Figure 1.**
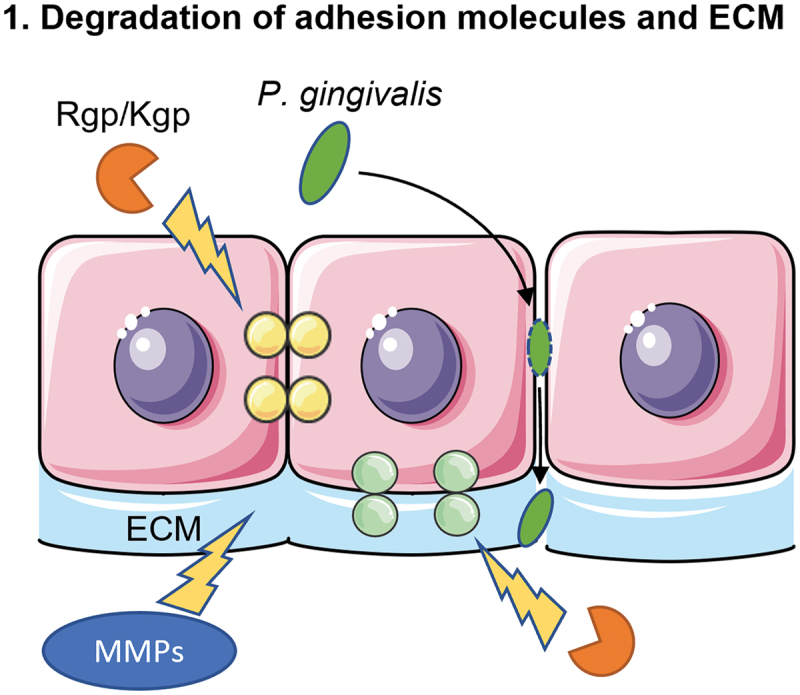
Schematic overview of the first mechanism of translocation. *P. gingivalis* secretes proteolytic enzymes known as gingipains (Rgp/Kgp) that degrade cell-cell adhesion molecules (yellow) and adhesion molecules (light green) that connect the cell with the extracellular matrix (ECM). In addition, *P. gingivalis* stimulates the fibroblasts to produce matrix metalloproteases (MMPs) that can degrade ECM molecules. These three effects combined weaken the integrity of the epithelial barrier, which allows *P. gingivalis* to travel between cells into deeper layers of the tissue.

**Figure 2.**
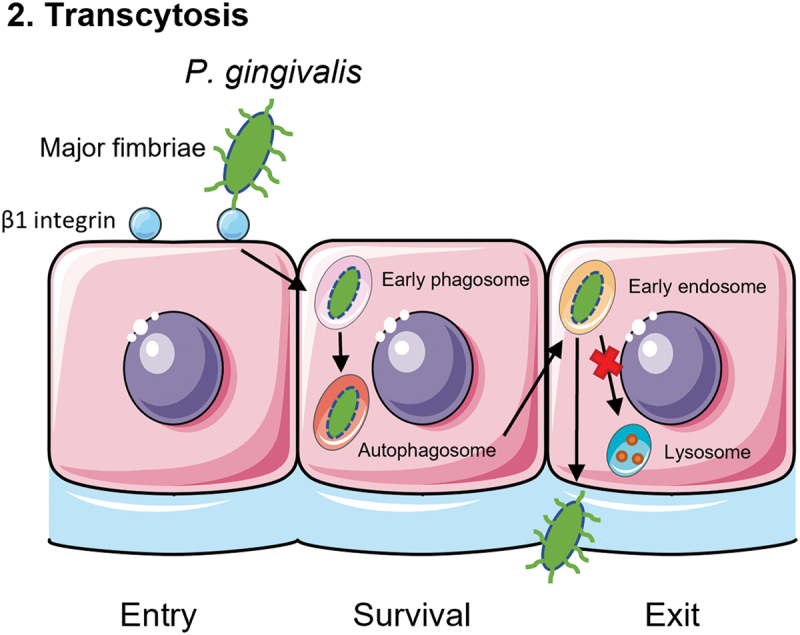
Schematic overview of the second mechanism of translocation. The major fimbriae of *P. gingivalis* adhere to the β1 integrin receptor of the epithelial cells. This will lead to entry of the bacterium into an early phagosome. In order to survive within the cell, *P. gingivalis* makes use of the autophagy pathway of the epithelial cell to prevent being transferred to lysosomes, where *P. gingivalis* would be killed. Lastly, the bacterium exits the cell via the endocytic recycling pathway.

**Figure 3.**
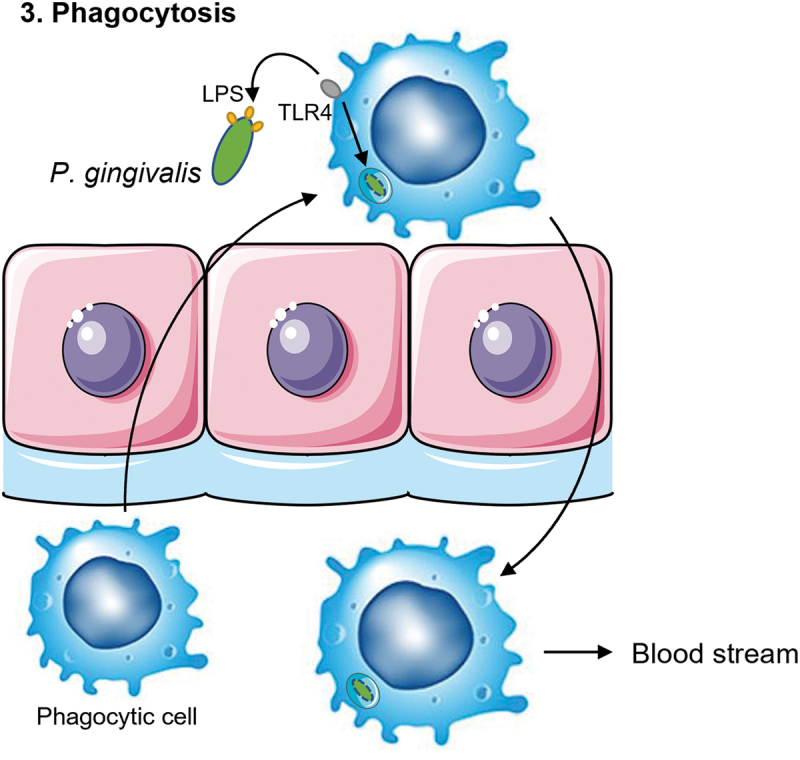
Schematic overview of the third mechanism of translocation. *P. gingivalis* is recognized by phagocytic cells such as macrophages, monocytes or dendritic cells. Toll-like receptor 4 (TLR4) recognizes the lipopolysaccharide (LPS) of *P. gingivalis* and subsequently phagocytoses the bacterium. Then, the phagocyte will travel back to the blood stream with *P. gingivalis* inside, as a ‘Trojan Horse’ mechanism.

**Figure 4.**
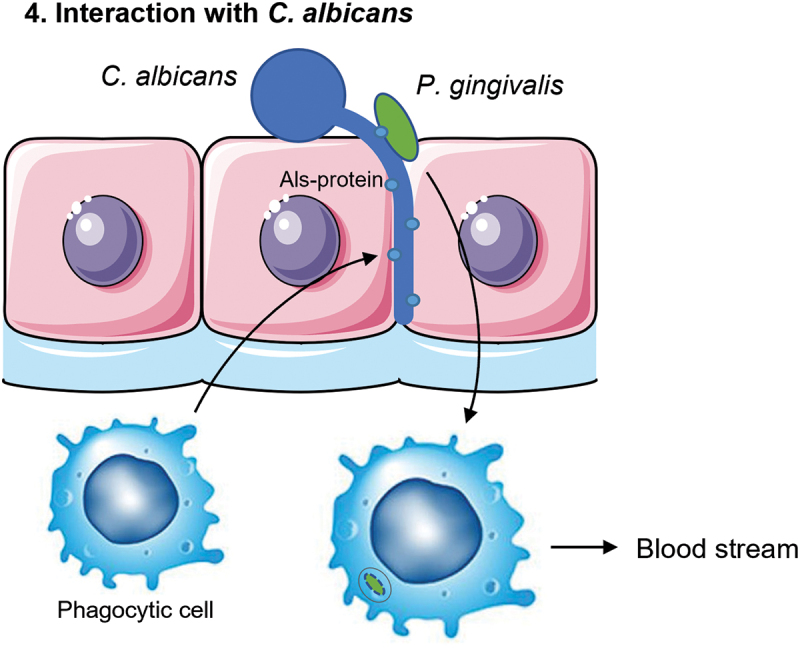
Schematic overview of the fourth mechanism of translocation. *C. albicans* can form invasive hyphae that insert themselves between the epithelial cells. *P. gingivalis* can adhere to *C. albicans* via the Als3 protein on the hyphae of the fungi. Phagocytic cells such as macrophages are attracted to *C. albicans*. This allows for the phagocytes to come in close contact to the bacterium and take up *P. gingivalis* to travel back into the blood stream.

**Figure 5.**
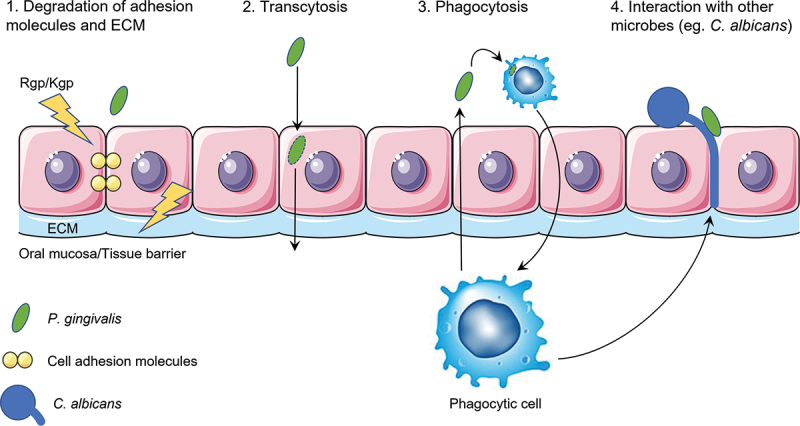
A schematic overview of the four putative mechanisms for *P. gingivalis* to translocate the oral mucosa or the endothelium of blood vessels. First, proteolytic enzymes known as gingipains (Rgp/Kgp) are secreted by *P. gingivalis* (green) and degrade cell-cell adherence molecules (yellow circles) and the extracellular matrix (ECM; light blue). The structural integrity of the oral mucosa is weakened so that *P. gingivalis* is able to pass in between the cells. Second, *P. gingivalis* can enter the cell after adherence and subsequent endocytosis and exit the cell on the other side of the epithelial layer. *P. gingivalis* needs to have a mechanism to be able to survive within the cell and not be degraded by phagolysosomes. It can then travel from cell to cell, migrating deeper into the tissue, eventually reaching the basal membrane, and finally the endothelial cells that line the blood vessels. Third, phagocytic cells of the host are able to pick up *P. gingivalis* within the tissue and transfer it over the endothelial barrier. They can then travel back into the bloodstream taking the bacterium with them. Again, *P. gingivalis* needs to have a mechanism to survive degradation by the phagocyte. Fourth and lastly, interaction with other microbes such as *C. albicans* (blue) could allow for travel across the oral mucosa due to the hyphae of *C. albicans* that can insert themselves between cells. *P. gingivalis* is able to attach to these hyphae. Macrophages may also play a role in this mechanism as they are attracted to the hyphae of *C. albicans* and can phagocytose the attached *P. gingivalis*.

The aim of this review is to describe potential mechanisms allowing translocation of *P. gingivalis* over the oral mucosa and disseminate into the bloodstream. It is important to understand these mechanisms as it might uncover possible targets for the prevention of these diseases. Based on research into *P. gingivalis* invasion and/or translocation, four different mechanisms could be identified by which *P. gingivalis* can translocate the oral mucosa barrier and endothelial linings, based on known mechanisms of either *P. gingivalis* or other microbes. These four mechanisms are separately described in this review, each with a detailed schematic depiction (Figures 1–4). A more simplified overview of all four mechanisms is shown in Figure 5.

## Mechanisms

### Degradation of adhesion molecules and extracellular matrix

The oral mucosal barrier can be disrupted by *P. gingivalis*-mediated degradation of the cellular adhesion molecules and extracellular matrix (ECM) (Figure 1). The structural integrity of the tissue is compromised after such proteolysis, creating space for the bacteria to penetrate deeper into the tissue. *P. gingivalis* is able to degrade cell-cell junction complexes Occludin, E-cadherin and β1-integrin between epithelial cells [24]. In addition, using a monolayer of Madin-Darby canine kidney (MDCK) cell line it was found that *P. gingivalis* decreases the transepithelial resistance (TER). The bacteria were also found to be translocating the monolayer of MDCK cells [24]. This suggests that *P. gingivalis* is able to invade the connective tissues *e.g*. by degrading the epithelial tissue structure and travelling between the cells. This allows the spread of the bacteria and may result in dissemination into the blood. It is unsure whether MDCK cells translate well to the oral mucosa but this study does suggest that *P. gingivalis* is able to invade epithelial barriers.

Following entry of the body by passing through oral mucosa layers, the bacteria would need to pass the endothelium as well to reach the bloodstream. It was found that gingipains are involved in the detachment and apoptosis of endothelial cells [25]. Kinetics of cell adhesion molecule cleavage differed between epithelial and endothelial cells. Gingipains induce cleavage of the adhesion molecules vascular endothelial cadherin (VE-cadherin), neural cadherin (N-cadherin) and integrin β1, as well as E-selectin and platelet endothelial cell adhesion molecule-1 (PECAM-1) [25,26].

When the adhesion molecules that facilitate the interaction between epithelial cells and the ECM are degraded, the structural integrity of the epithelium is affected. This may facilitate the penetration of the bacteria deeper into the tissue. The gingipains secreted by *P. gingivalis* are also known to degrade molecules of the ECM, such as fibronectin and tenascin-C. This was found via fragmentation of fibronectin and tenascin-C by purified proteases, observed in a SDS-PAGE gel and via immunofluorescence staining [27,28]. Using a complex three-dimensional models of engineered human oral mucosa (EHOM) it was illustrated that *P. gingivalis* is able to penetrate into the tissue by degradation of components of the basement membrane [21]. EHOM is composed of epithelial cells and fibroblasts from healthy human biopsy specimens. Using transmission electron microscopy on this model, it was found that wild-type *P. gingivalis* is able to penetrate into deeper structures of the tissue as compared to gingipain-deficient mutants of *P. gingivalis*.

Outer membrane protein A-like proteins are known to be involved in ECM binding by bacteria. *P. gingivalis* can adhere to ECM via outer membrane proteins Pgm6 and Pgm7 they form a heterotrimer (Pgm6/7) [29]. In addition, *P. gingivalis* is able to adhere to ECM molecule fibronectin via its fimbriae and the affinity of binding is enhanced when fibronectin is degraded by gingipain-like proteases [30]. Thus, gingipains contribute directly and indirectly to adherence and colonization of epithelial surfaces such as the oral mucosa.

*P. gingivalis* is also indirectly involved in the degradation of the extracellular matrix. Studies have shown that *P. gingivalis* supernatant stimulates the release of matrix metalloproteases (MMPs) in human gingival fibroblasts on the mRNA level. In addition, the tissue inhibitor of MMPs (TIMP) expression in reduced [31,32]. These MMPs are known to degrade various extracellular matrix proteins such as collagen.

Not only the gingipains are important virulence factors that have an effect on the structural integrity of epithelial and endothelial tissue, this process also occurs indirectly via the immune system. *P. gingivalis* can dysregulate host immune responses to benefit other pathogens. For example, *P. gingivalis* can cleave or induce cross-talk in immune cell receptors or suppress the release of cytokines [33,34]. Dysbiosis of the microbial community occurs which causes inflammation in the periodontium [34]. This inflammation could enhance the epithelial cell damage and vascular leakage, which increases the ability of *P. gingivalis* to translocate.

### Invasion of epithelial and endothelial cells (Transcytosis)

Transcytosis is a pathogen invasion process, in principle involving three essential phases: entry, survival and exit [35]. Figure 2 shows a detailed overview of the mechanism. Entry of the pathogen can occur either through active or passive mechanisms. In the active process, the bacteria attach to the surface of a host cell. This can occur either by the interaction of bacterial surface proteins with proteins on the host cell, or the insertion of effectors by the bacterium into the membrane of the host cell [36]. Then, the bacterium is engulfed by the cell and an intracellular vesicle will form which contains the pathogen. This process is dependent on reorganization of the cytoskeleton of the invaded cell [36–38]. The passive mechanism involves phagocytosis by the host cell [35]. Only professional phagocytes such as macrophages can perform this mechanism with high efficiency. They are a group of cells that are specialized to remove microorganisms, whereas non-professional phagocytes such as epithelial cells cannot ingest microorganisms [39].

After entry, the bacterium needs to have a mechanism to survive within the cell. Generally, when an epithelial cell is invaded by a pathogen, the host cell will fuse the pathogen-containing intracellular vesicle with lysosomes in order to kill the invading pathogens [40]. Several mechanisms exist for evasion of this process, each invading pathogen species has its own survival strategy. For example, the pathogen can survive by evading fusion with a lysosome or resisting degradation once inside the phagolysosome [36,38]. Lastly, the bacterium needs to be able to exit the cell on the other side into deeper structures of the tissue. Three main strategies are described by which an intracellular pathogen can exit the cell: Induction of programmed cell death of the host, active destruction of the host cell, and membrane-dependent exit without destruction of the host cell [41]. By travelling from cell to cell, bacteria can travel deeper and deeper into the tissue and can eventually enter the bloodstream.

The first step of transcytosis, cell entry by *P. gingivalis*, has been researched extensively. Already two decades ago it was discovered that this bacterium is able to rapidly invade primary gingival epithelial cells [42,43]. Adherence and subsequent invasion of *P. gingivalis* into gingival epithelial cells is mediated by binding of major fimbriae to β1 integrin receptor which induces the phosphorylation of paxillin (integrin-clustering associated protein) [44,45]. While *P. gingivalis* is generally considered to be a non-motile bacterium, recent research has shown that the bacterium is able to translocate over surfaces when sandwiched between two surfaces [46]. This mechanism might occur when *P. gingivalis* is located between different epithelial cells. Possibly, this newfound mobility supports the potency of interaction with β1 integrin, in order to adhere and subsequently invade the tissue cell.

After invasion of the epithelial cell layer, the bacterium will enter the extracellular matrix of the connective tissue, and subsequently, it will encounter the endothelial cells that line the blood vessels. These vascular endothelial cells can also be invaded by *P. gingivalis*. However, they are less susceptible to invasion than oral epithelial keratinocytes [26]. This process was found to be gingipain-dependent. It is suggested that the intracellular *P. gingivalis* are enclosed in vesicles, which leads to the hypothesis that endocytosis is the mechanism by which *P. gingivalis* was internalized [26].

For the second step in transcytosis, survival, *P. gingivalis* is dependent on a mechanism to evade degradation via lysosomes. It is suggested in multiple studies that this is achieved by trafficking via autophagic pathways [37,47,48]. After internalization into gingival epithelial cells, *P. gingivalis* is incorporated into early phagosomes. Then, *P. gingivalis* travels to autophagosomes of the host cell to provide a nutrient-rich environment in which the bacterium can survive. Here, *P. gingivalis* can somehow prevent lysosomal hydrolases from entering the autophagosome and thereby evading degradation. When the autophagic pathway was inhibited, *P. gingivalis* was transferred to phagolysosomes where they were degraded [47].

The third and final step for *P. gingivalis* to disseminate into the blood stream by transcytosis is for the bacterium to exit the cells. It was suggested that exit of *P. gingivalis* from infected cells occurs via the endocytic recycling pathway [49]. The endocytic recycling pathway is a dynamic system inside the cell that is responsible for sorting and exporting membrane components. First, the internalized cargo is transported to the early endosome by membrane fusion. There it is sorted and will either be transported for degradation by lysosomes or returned to the outer membrane of the cell [50].

When *P. gingivalis* invades a gingival epithelial cell, it remains inside an intracellular vesicle. This vesicle will then fuse with the early endosome. It is suggested that *P. gingivalis* has a mechanism by which it evades the transfer to the lysosome but instead will enter the recycling pathway back to the cell membrane [51]. Intracellular *P. gingivalis* was found to be co-localized with transferrin receptor, Rab11 and RaIA, which are components of the recycling pathway. When the recycling pathway was inhibited by knockdown of *Rab11* or *RaIA*, exit of *P. gingivalis* from gingival epithelial cells was reduced. It was also found that exit of the bacterium from gingival epithelial cells depended on actin polymerization, lipid rafts and microtubule assembly. These processes are also associated with the recycling pathway, suggesting that this pathway and exit of *P. gingivalis* from gingival epithelial cells might be connected [49].

### Phagocytosis

Periodontitis is characterized by systemic inflammation of the gingiva [52]. This attracts immune cells, among which are professional phagocytes such as monocytes and macrophages [53]. Studies have shown that macrophages are found in higher abundance in gingival biopsies of gingivitis and periodontitis patients as compared to healthy gingiva [54,55]. Professional phagocytes such as macrophages are highly specialized to engulf and destroy pathogens. However, some pathogens can evade killing by the macrophage after phagocytosis and use the macrophage as an entry into other tissues of the host. This strategy is known as a ‘Trojan Horse’ mechanism and is used by *Bacillus anthracis*, for example [56]. The phagocyte takes up the pathogen in the periphery (lungs, intestine or oral cavity) and travels into the bloodstream (Figure 3). Inside the phagocyte, the pathogens are protected from the host immune system and can multiply. The phagocyte will travel to another organ where the pathogen can exit the phagocyte and causes an infection in that organ. This mechanism can also lead to infection of the brain, when the phagocytes pass the blood–brain barrier [57].

This Trojan Horse mechanism could also be a way for *P. gingivalis* to disseminate into the blood. One major condition for this to occur is that *P. gingivalis* has the ability to survive within the phagocyte, and escape effectively. Research has shown that *P. gingivalis* was able to effectively invade and viably escape macrophages [58]. This was observed with a phagocytosis assay: THP-1 derived macrophages with M0 (naive) or M1 (pro-inflammatory) phenotype and RAW264.7 cells were infected with two different strains of *P. gingivalis*, *Streptococcus gordonii* or *Escherichia coli* and then the extracellular bacteria were killed with antibiotics. After 24 hours (long enough to allow for potential phagolysosome killing of bacteria) the cells were lysed and the number of intracellular bacteria that survived were determined by counting colony forming units (CFU). The efficiency of escape was determined by collecting supernatant after killing the extracellular bacteria, instead of lysing the cells. It appeared that strain W83 in particular was very effective in invading and escaping THP-1 derived macrophages and RAW 264.7 cells. Strain ATCC 33,277 of *P. gingivalis* also showed invasion and escape, significantly more than other microbes tested (*S. gordonii* and *E. coli*) [58]. Active invasion of phagocytic cells was also shown by *P. gingivalis*. Here, the invasion of macrophages by *P. gingivalis* is dependent on the major fimbriae. This was found in a study using a fimbriae deficient mutant of *P. gingivalis* (DPG3) that adherence and invasion of THP-1 cells was compromised, compared to wild type [59]. The mechanism behind this process is not completely clear and more research is needed to fully unravel this process.

Macrophages can polarize into pro-inflammatory M1 or anti-inflammatory M2 phenotypes. The M1 macrophages are responsible for phagocytosis and subsequent killing of bacteria, and production of pro-inflammatory cytokines. On the other hand, M2 macrophages are involved in the reduction of inflammation, wound healing and tissue repair [60,61]. The anti-inflammatory M2 macrophages might be responsible for the survival of bacteria after phagocytosis and thereby act as the Trojan horse to bring bacteria into the blood stream. Studies have shown that bacteria such as *Chlamydia pneumoniae* and *Brucella melitensis* are more likely to survive in M2 macrophages compared to M1 macrophages [62,63]. The same was shown for *P. gingivalis*: they can survive in naïve and M2 macrophages for 24 hours, but not in M1 macrophages [64]. This was found by determining the number of bacteria that are taken up by the macrophages after 1 hour and relating these to the number of bacteria that were still present inside the macrophages after 24 hours using the phagocytosis assay described above.

A capsule-deficient mutant of the *P. gingivalis* strain W50 was used in order to investigate the effect of the capsule on host response. Phagocytosis was determined by flow cytometry of mouse bone-marrow derived macrophages and dendritic cells after they were exposed to FITC-labeled bacteria. Survival was determined by lysing the cells, plating and counting the CFU. The capsule-deficient mutant of *P. gingivalis* was phagocytosed much more by the macrophages and dendritic cells than the wild-type strain. In contrast, no surviving capsule-deficient *P. gingivalis* were found in the phagocytes, whereas the wild-type did survive. In addition, it was found by ELISA that the capsule of *P. gingivalis* can aid in reducing the inflammatory response of the host [65].

Lipopolysaccharide (LPS) is expressed on Gram-negative bacteria and can activate macrophages via Toll-like receptor 4 (TLR4) to phagocytose the bacteria [66,67]. It was found that *P. gingivalis* LPS only weakly activates macrophage polarization but does induce a release of cytokines and chemokines that attract other immune cells [68].

In addition to macrophages, dendritic cells (DC) are also phagocytic cells that could be involved in the translocation of *P. gingivalis* over the mucosal barrier. Already over two decades ago, it was found that *P. gingivalis* can be internalized into DCs and activate them after penetration into the gingival epithelium [69]. The minor fimbriae of *P. gingivalis* can target DC-Specific Intercellular adhesion molecule-3-Grabbing Non-integrin (DC-SIGN) and enter the cell. DCs in periodontitis lesions can become activated and will travel to the lamina propria, which is highly vascularized. It is hypothesized that upon infection, *P. gingivalis*-containing DCs travel through the lamina propria to the bloodstream which will allow the bacterium to reach atherosclerotic plaques [70]. It was found that *P. gingivalis* is able to survive within monocyte-derived DCs as well, after uptake via DC-SIGN. The bacterium uses its Mfa-1 fimbriae to evade antibacterial autophagy and lysosome fusion. However, the activation of TLR2 can promote killing of the bacterium, which is stimulated by the major fimbriae FimA of *P. gingivalis* [71].

### Interaction with other microbes

The fourth and final mechanism of actively passing penetrating host tissue barriers is via interaction with other (oral) microbes such as *Candida albicans* (see Figure 4). This mechanism is particularly relevant for passing the oral mucosa. *C. albicans* is a polymorphic fungus, i.e. it can grow as yeast in a spherical appearance or it can adapt the pseudohyphae or hyphae morphology, and it is a common colonizer of the oral cavity. In healthy individuals, *C. albicans* grows mostly as yeast without causing tissue damage. Formation of hyphae allows for invasion and destruction of epithelial cells. Many oral bacteria interact with *C. albicans* and some utilize the tissue invading hyphae to cross the oral mucosa barrier [72]. For example, *Staphylococcus aureus* is able to adhere to hyphae of *C. albicans*. This adhesion is mediated by the Als3 protein, a member of the agglutinin-like sequence (Als) family of *C. albicans*. In a murine model of oral candidiasis, interaction with *C. albicans* allowed co-colonizing *S. aureus* to invade deeper into the tissue and disseminate into the blood [73,74]. There was no dissemination when the mice were infected with *S. aureus* alone. However, further investigation showed that adhesion of the bacterium does not progress with the hyphae, meaning *S. aureus* cannot be actively transported across the oral mucosa in this manner [75]. Another mechanism must be in place in order for *C. albicans* to increase *S. aureus* dissemination. This has led to the hypothesis that *S. aureus* can also disseminate through the previously described Trojan Horse mechanism by surviving inside macrophages after phagocytosis. It has been observed that macrophages are highly attracted to *C. albicans* hyphae. This attraction is mediated by the interaction between the β-glucan on *C. albicans* hyphae and dectin-1 on macrophages [76,77]. This attraction leads to an increase in phagocytosis of *S. aureus* that are adhering to the hyphae of *C. albicans* and could explain why S. aureus disseminates into the blood much easier in the presence of *C. albicans* [74,75].

The same mechanism could also occur for *P. gingivalis*. Studies have shown that *P. gingivalis* is able to interact with and can adhere to *C. albicans* [78,79]. This adherence occurs in a similar way as the adherence of *S. aureus* to *C. albicans*, namely in an Als3-dependent manner [79–81]. In addition, *C. albicans* enhances *P. gingivalis* invasion of human gingival epithelial cells and fibroblasts [82]. Seeing that *P. gingivalis* is able to survive within macrophages (as described previously), the interaction with *C. albicans* could be a plausible component of the mechanisms of *P. gingivalis* to disseminate into the bloodstream [58]. However, a more recent study found that immune responses in THP-1 derived macrophages were reduced when exposed to a mixed biofilm of *P. gingivalis* and *C. albicans*, compared to supernatant of bacterial-only biofilms. This was measured by ELISA of cytokines and chemokines IL-1β, IL-8 and TNFα. While IL-1β production was increased, production of IL-8 and TNFα was significantly reduced when exposed to the mixed biofilm. These results were supported with an *in vivo* model using C57BL/6 mice, where it was found that the mice survived longer when infected with both species compared to infection with *P. gingivalis* alone. In addition, the kidney and spleen of the mice were found to be infected with *P. gingivalis* to a higher extent when the mice were infected with *P. gingivalis* alone, as compared to mixed species infection. It is suggested that this is due to a lower inflammatory response, as it was also found that dual-species infection leads to a reduced elastase and myeloperoxidase production, which are both neutrophil granular enzymes associated with inflammation [83]. The suggestion that *C. albicans* protects *P. gingivalis* from host immune responses would support the theory that *C. albicans* is involved in *P. gingivalis* survival, but the study does not strengthen the hypotheses that *C. albicans* is involved in *P. gingivalis* dissemination, and the role of macrophages in this process.

Next to *C. albicans*, there is also evidence that the bacterium *Fusobacterium nucleatum* is involved in the ability of *P. gingivalis* to invade the tissue. *F. nucleatum* is also commonly found in subgingival plaque and is considered to be part of the ‘orange complex’ in the pyramid of Socransky [6]. The influence of *F. nucleatum* on cell invasion by *P. gingivalis* was observed using antibiotic protection assays, as described previously in this review. In this study, the cell line Ca9–22 was used as a model for the gingival epithelium and Human aorta endothelial cells (HAEC) were used as a model for the endothelium. Invasion into both cell types by *P. gingivalis* was significantly enhanced in the presence of *F. nucleatum*. It is suggested that coaggregation of the two bacterial species may influence the expression of virulence factors. However, the exact molecular mechanism of how *F. nucleatum* enhances *P. gingivalis* invasion into gingival epithelial and endothelial cells remains to be elucidated.

## Discussion

*P. gingivalis* has been associated with multiple systemic diseases, in addition to oral disease. For this to occur, the bacterium needs a mechanism to travel from the oral cavity into the bloodstream so that it can reach other organs. Easy access into the blood stream would be via passive entry by bleeding gums, which is common in periodontitis. Considering that *P. gingivalis* can be found in other organs unlike many other oral microorganisms, it is plausible that *P. gingivalis* also has an active mechanism to enter the blood stream through epithelial layers. In addition, active translocation from the blood circulation is needed to reach other organs in the body such as the brain. Four active mechanisms have been proposed by which *P. gingivalis* can cross tissue barriers such as the oral mucosa.

Most components of the described active mechanisms have been tested in in vitro experiments such as in monolayer tissue cultures, with some studies using primary gingival epithelial cells, but also studies using cells that are not from the oral epithelial origin, such as kidney cells [24]. Apparently, within the limitations of these systems which are not always representative for the oral mucosa; there could be interplay between the different cell types found in the tissue and extracellular matrix surrounding the cells. Therefore, such studies should first of all be repeated on genuine oral epithelial cells, in order to establish in a pure culture system whether for instance *P. gingivalis* disrupts cell-cell adhesion structures here as well. To study the effects of *P. gingivalis* infection on cell-cell adhesion or the release of certain factors such as cytokines, these monolayer cultures are sufficient. However, in order to study penetration into the deeper tissue layers and the interaction between the different cells of the tissue layers, these monocultures would not be sufficient. Use of a more sophisticated three-dimensional model of the oral mucosa could give more insight in the effects of *P. gingivalis* on the integrity of the tissue structure. There are already some studies where such a model is being developed, which can be used to observe bacterial penetration into host tissue [84,85].

In addition, the studies using phagocytotic cells such as macrophages generally make use of THP-1 derived macrophages. This is a monocytic cell line derived from human leukemic cells. Likely, under culturing conditions these cells will phenotypically deviate from primary monocytes and macrophages. However, studies on the phagocytosis of *P. gingivalis* using macrophages that are derived from primary monocytes are currently lacking in literature.

In one of the studies where the degradation of adhesion molecules was studied, it was also found that the trans-epithelial resistance decreases in the presence of *P. gingivalis*. This shows that the barrier functionality of the epithelium is affected and might therefore allow *P. gingivalis* to penetrate [24]. This suggests that the mechanism for *P. gingivalis* to invade the deeper epithelium occurs via the intercellular pathway rather than intracellular, to eventually invade the blood capillaries. This was previously also suggested as the most likely entry by *P. gingivalis* in a review covering various transepithelial models to study host–pathogen interactions [86]. Cell invasion might then preferably be used by *P. gingivalis* as a mechanism for evasion of the host immune system and resistance from antibiotic treatment by hiding inside the epithelial and endothelial cells, rather than a way to transport itself further into the tissue [26]. A combination of pathways might also occur in order for *P. gingivalis* to more effectively cross the oral mucosa barrier. For instance, the involvement of *C. albicans* might enhance this mechanism of degrading adhesion molecules, as the hyphae are known to cause tissue damage and penetrate into the oral epithelium. On the other hand, macrophages provide both protection from other host immune responses and antibiotic treatment as well as a way to cross the oral mucosa, which would suggest it would be easiest mechanism for *P. gingivalis* to travel to the bloodstream. It is also a possibility that *P. gingivalis* actively infiltrates the macrophages, instead of them being phagocytosed, combining transcytosis with the ‘Trojan Horse’ mechanism. However, only a small number of bacteria would be able to travel this way as it was observed *in vitro* that 2–3% of bacteria invade macrophages and 10–20% of these survive [64]. Of course, the attraction of macrophages to *C. albicans* might increase the amount of phagocytosis of *P. gingivalis* that are adhered to *C. albicans* as opposed to *P. gingivalis* alone. Very little research has been done for the third and fourth mechanism of translocation described in this review however, and more research might elucidate further how *P. gingivalis* penetrates the oral mucosa (and possibly other tissue barriers) and whether a combination of these mechanisms occur (see Figure 5).

It has to be noted that not all of the discussed mechanisms are experimentally supported, especially those on phagocytosis by macrophages and the involvement of *C. albicans*. These are still hypothetical and research needs to be done to confirm whether these mechanisms can actually occur. However, in the case of *C. albicans*, a lot of research has been done on the interaction between *S. aureus* and *C. albicans* [73–75,80]. Unpublished results in our group indicate that some aspects of this interaction are also apparent for *P. gingivalis*. There are some studies on the interaction between *P. gingivalis* and *C. albicans*, however this is more limited [78,81,83]. In this review it was hypothesized that *P. gingivalis* might have a similar interaction with *C. albicans* as *S. aureus*. That *P. gingivalis* can adhere to *C. albicans* in a similar way as *S. aureus* has been observed before, but the interaction with macrophages still has to be proven.

In the case of being a risk factor for Alzheimer’s disease, in addition to the oral mucosa the bacterium would also need to pass the blood–brain barrier (BBB). Being composed of many cell types, the BBB is another challenging structural and functional barrier for microorganisms. The vessels in the brain do not contain any pores and its cells are tightly adhered together [87]. Transport across the barrier is regulated by specific transport proteins. This makes the blood–brain barrier highly selective and it is specialized to protect the brain against pathogens and toxins [88,89]. However, infection of the brain has been known to occur for various microorganisms. Multiple reviews about bacterial translocation of the BBB describe three of the four mechanisms described in the current review, including: disruption of adherence molecules, transcytosis and the ‘Trojan Horse’ mechanism via macrophages [90–92]. Research into the blood–brain barrier is challenging as it is difficult to represent this barrier *in vitro*.

Unraveling the mechanism by which *P. gingivalis* may cross rigid, protective structures such as the oral epithelium, will provide insight for eventual dissemination into the blood, but equally important: similar mechanisms could occur for *P. gingivalis* to travel from blood into tissue, as similar barriers have to be crossed. It is essential to find possible targets to prevent the bacterium to affect other organs in the body leading to systemic diseases such as atherosclerosis and Alzheimer’s disease. For example, a gingipain inhibitor could be used for periodontitis patients in order to reduce the dissemination of *P. gingivalis* into the bloodstream and its subsequent entry into other organs. Not only *P. gingivalis* but other periodontal pathogens are associated with systemic diseases [11–13]. Unraveling the dissemination mechanisms for *P. gingivalis* might open some doors for research into other oral pathogens that might have similar mechanisms. In addition, these oral pathogens might strengthen each other’s dissemination mechanisms, such as the interaction between *P. gingivalis* and *C. albicans*. This emphasizes the importance of a balanced oral microbiome and how disbalance can have an impact on systemic health.
